# SKIP: emotional well-being intervention

**DOI:** 10.1192/bji.2020.7

**Published:** 2020-05

**Authors:** Natalie Cook

**Affiliations:** Adult Mental Health, Humber NHS Foundation Trust, Willerby, UK. Email: natalie.cook@york.ac.uk

**Keywords:** Education and training, low- and middle-income countries, transcultural psychiatry

## Abstract

SKIP (Students for Kids International Projects) is a student-led global health charity; each university branch partners with a local non-governmental organisation in their branch country, where they run interventions identified by the local community. Research in these countries has identified an educational need for interventions around emotional well-being. In this article, we reflect on the process of creating culturally appropriate educational resources for children and young people in low- and middle-income countries, to be delivered by non-professionals. SKIP has a Research Ethics Policy. No external ethics approval was required.

Since 2008, I have volunteered with a student-led charity known as SKIP (Students for Kids International Projects). As a first-year medical student, I attended an information evening at Hull York Medical School, where I heard about a volunteering project with a community in Tanzania. I signed up and the following summer worked alongside a local non-governmental organisation (NGO) called MYODA to run health promotion sessions, sexual health education and disability awareness groups.

SKIP's vision is two-fold: first, for all children to be cared for and supported in accessing the basic rights of health, welfare and education within their communities by developing sustainable community-based projects; second, to develop globally aware students who can advocate for local and international health progress as future professionals. We currently have SKIP branches in 11 UK universities, each of which partners with a local NGO in their project country.

Little did I know that my decision to volunteer that summer would have a massive influence on the next decade of my life. Fast-forward to 2019, I am now a SKIP trustee, currently involved in the development of an emotional well-being intervention.

The idea of developing the intervention came from SKIP Newcastle volunteers in Cambodia, who conducted some research into mental health issues over summer 2018. The volunteers organised a meeting at the local health centre, where they spoke to the centre director and a local midwife. In this meeting, they discussed local healthcare provision, local understanding of distress and stigma issues. They found that people in the local community did not really seek medical help for stress or feeling sad but tended to seek support from their family. Both professionals felt that talking about mental health with the community would be well received. The volunteers also met with a member of the American Peace Corps who had been living with the community for 2 years. They felt that stress in the local community was amplified by financial concerns, alcohol, political instability and historical national trauma related to Khmer Rouge.

After these meetings, SKIP volunteers led two focus groups in the community, with 72 attendees (55 women and 17 men). Attendees were given written questions; those unable to read were offered the chance to speak to the volunteers separately. More than 68% of attendees could identify members of their community who felt sad or stressed. There were many different issues that resulted in these emotions, including difficulties with family life (domestic violence, infidelity, arranged marriage and bereavement), financial stress, physical ill-health, loneliness, alcohol excess and alcohol-related violence, as well as erratic weather causing events such as flooding. Adults felt that children were affected by hunger, exams, bullying and not having enough money. When asked what people do when they feel sad or stressed, attendees said they found others to talk to in the community, read a book, went for a walk or watched comedy.

Reflecting on the effects of these emotions on functioning, people identified that they make it harder to go to work and have an effect on family and friends. The consensus of the focus groups was that implementing emotional well-being lessons in local schools would be welcomed and seen as beneficial.

On their return from the summer project, SKIP Newcastle agreed to collaborate with another branch, SKIP Glasgow, and some members of the National Committee to form a small working group. In this group, we identified a few initial tasks which were distributed throughout the team – these included gathering background information on Cambodia using the World Health Organization Mental Health Atlas, a literature search on interventions already existing in Cambodia, and a more general literature search looking for layperson-led interventions. From our research, we identified six NGOs working in this field in Cambodia, the largest being the Transcultural Psychosocial Organisation.

After the initial focus on Cambodia, which helped us determine the need, we began to generalise the research to other project countries. As all SKIP interventions must be evidence based, our next step was to develop research tools. We created a standardised needs assessment tool to gather information about the local community, resources and language/terminology used to communicate distress within the community, the idea being that branches should complete this tool before developing the intervention with their partner NGO to ensure it matches the needs of the community.

At this stage, we began to have discussions about appropriate language for this intervention. We eventually decided to avoid using any medical, westernised terminology (such as mental health or depression), focusing instead on emotions and individual experiences. Terminology will be adapted following initial research, depending on the project country; in this way, it is hoped that the interventions will be more acceptable and understandable to the local community.

As a group, we spent some time creating lesson plans targeted at different age ranges, from junior school age to adult. The first couple of lessons are based around the theme ‘About Me’, to create a safe space to share information and talk about emotions. Activities include making an ‘All About Me’ flower or poster. The next few lessons cover ‘Understanding Emotions’, focusing on happy, sad, anger, worry and stress. Activities involve exploring emotions creatively through art and music, thinking about what these emotions might look like in other people through role-play and, for older children and adults, explaining the flight, fight or freeze model through scenarios.

The final couple of lessons focus on ‘Looking after Yourself’ – thinking about self-care and sources of support. Activities include thinking about helpful and unhelpful coping strategies (including alcohol and drugs), learning basic relaxation/mindfulness exercises and, for the older children and adults, talking about stigma surrounding worry and stress. To assess the effectiveness of these lessons, volunteers will conduct monitoring and evaluation exercises; these might take the form of short quizzes, focus groups and informal feedback.

Finally, in order for these interventions to be delivered successfully, project volunteers will need to be trained by our branch committees. Before they travel, volunteers will have time to test and adapt lesson plans depending on their project country and the available resources. To encourage a greater understanding of empathy, we are using a training technique based on ‘A Secret History’.^1^ In this method, groups are split in half – one half acting as the SKIP volunteer while the other is the community member. The purpose of the role-play is to explore a scenario where a person experiences distress and consider what both parties’ feelings and needs might be as the scenario evolves. The activity concludes with a discussion around individual reactions to stress, including stress buckets and various techniques to express empathy. Once they arrive on project, volunteers will also need to train translators so the lessons can be delivered effectively.

We hope that this intervention will continue to evolve and adapt to local needs over the next few years. The process of creating the intervention has been a learning curve, and I am sure that we will continue to face barriers. To increase the chances of the intervention being successful, we will have to ensure we work alongside our partner organisations and communities. In the past couple of months, two other branches have expressed interest in conducting needs assessments in their communities, and we hope the interventions will continue to expand over the next few years.
